# Supplementary figures and images for: Nonlinear Gap Junctions Enable Long-Distance Propagation of Pulsating Calcium Waves in Astrocyte Networks
Source: PLoS Comput Biol. 2010 Aug 26;6(8):e1000909. doi: 10.1371/journal.pcbi.1000909 (PMC2928752; doi:10.1371/journal.pcbi.1000909)

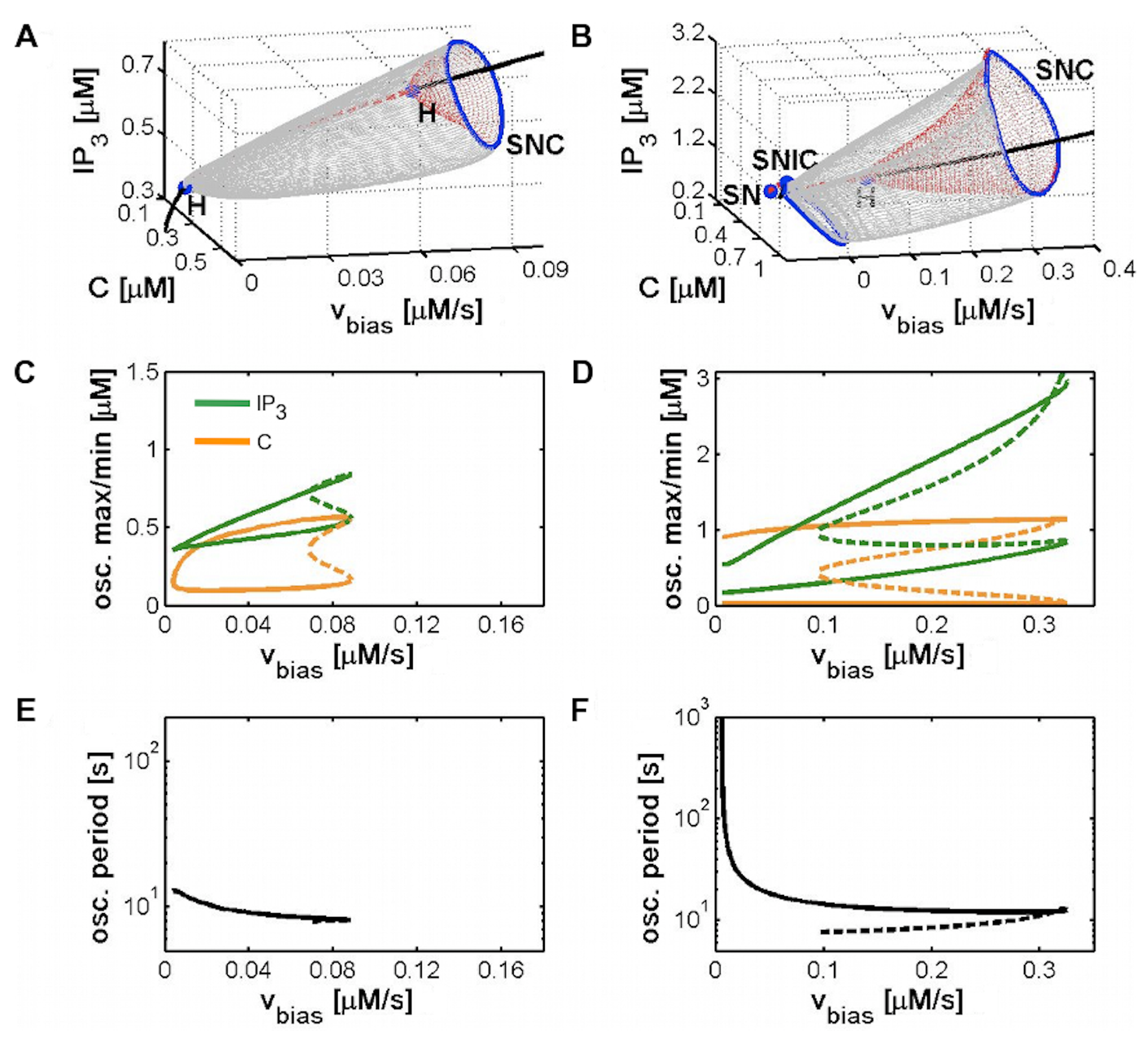

Supplement: Figure S1 — Bifurcation analysis of an uncoupled (i.e. isolated) ChI astrocyte for AFM (a, c, e) and FM (b, d, f) encoding regimes. (a, b) 3D-rendering of bifurcation surfaces in the state space. AFM oscillation amplitude (c) and period (e) are controlled by a supercritical Hopf (H) bifurcation and a saddle-node limit cycle (SNC) bifurcation respectively. Conversely in FM-mode, the occurrence of a saddle-node on an invariant circle (SNIC) bifurcation accounts for the rise of arbitrarily-small frequency Ca2+ oscillations (f) at almost constant amplitude (d). Legend: (a, b): black lines: stable fixed points; red dashed lines unstable fixed points; blue lines: bifurcating limit cycles; semi-transparent surfaces denote envelopes of stable (grey) and unstable (red) oscillations. (c–f): green: IP3; orange: Ca2+ (c) full lines: stable oscillations; dashed lines: unstable oscillations. Parameters as in Table 1. (4.70 MB TIF) [file pcbi.1000909.s001.tif]

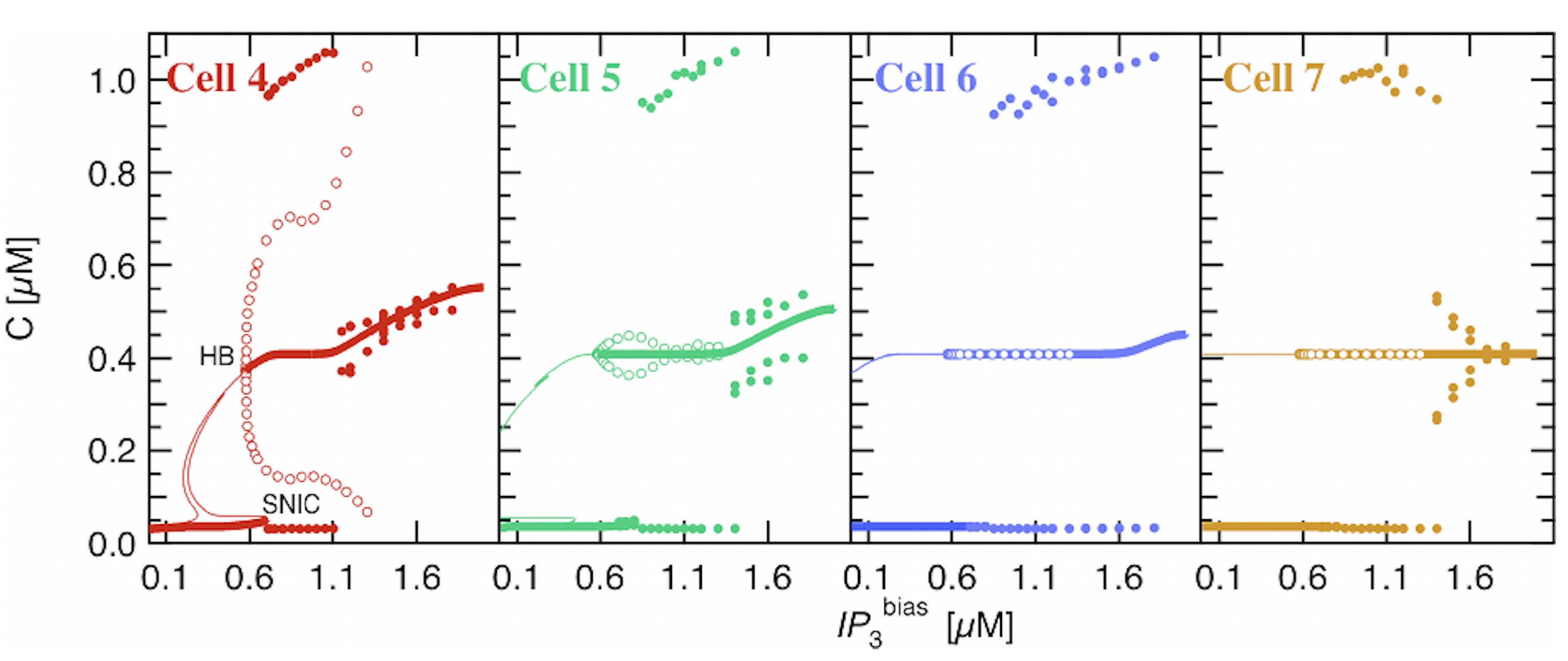

Supplement: Figure S2 — Bifurcation analysis of the astrocyte chain model for N = 7 FM-encoding cells with sigmoid coupling and reflective boundary conditions. Calcium concentrations at steady states are shown for the central (stimulated) cell (Cell 4) and for cells 5, 6 and 7. Although not apparent in the figure, for IP3 bias values larger than ≈0.8 µM, the stable oscillations become far more complex than in the isolated case. This is due to a very rapid cascade of period-doubling bifurcations, which yields extremely complex limit cycles (with numerous folds) that could not be precisely rendered in the figure (see also Section III.1.b). Moreover, for IP3 bias >1.1 µM, the amplitude of these limit cycles shrinks and numerical investigations evidenced the coexistence of multiple complex stable orbits. Legend: thin full lines locate unstable fixed points, and thick full lines stable one. Full (open) circles denote the envelopes of stable (unstable) limit cycles. Letters denote bifurcation type as in Figure SI1. (1.66 MB TIF) [file pcbi.1000909.s002.tif]

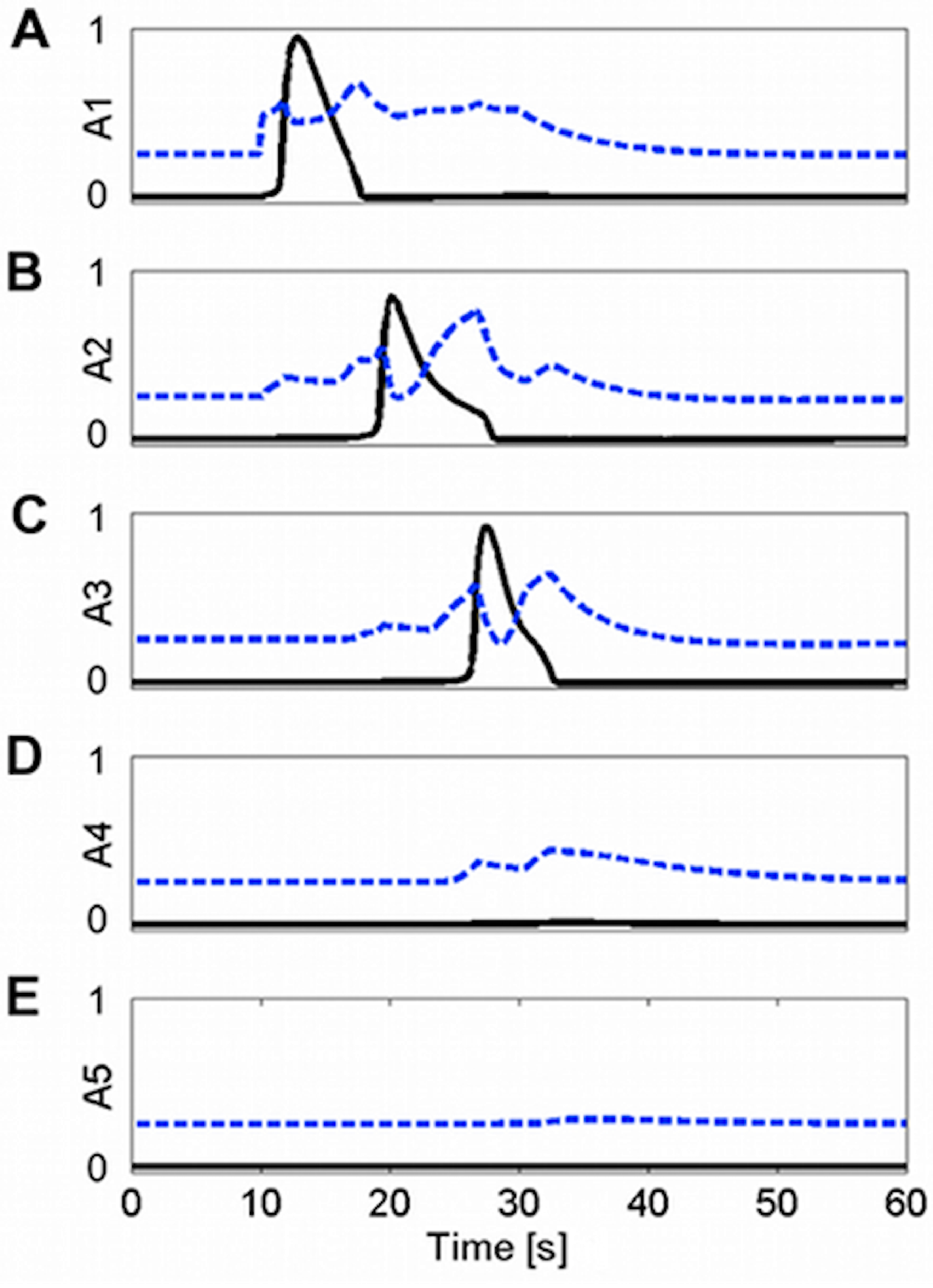

Supplement: Figure S3 — IP3-triggered CICR-mediated propagation of a pulsed Ca2+ wave within a chain of five FM ChI astrocytes (A1–A5). (a) An IP3 stimulation of constant intensity (IP3 bias = 0.8 µM) is applied to cell A1 from t = 10 s to t = 30 s. This increases IP3 concentration, thus triggering CICR from the ER and the generation of a Ca2+ pulse. (b) By means of communication through gap junctions, suprathreshold IP3 from A1 can diffuse to A2, triggering CICR there. The process is essentially regenerative so that a Ca2+ pulse almost identical to the original one can be observed in the arrival cells. (c) As soon as the IP3 influx to one cell from its neighbors is not sufficient to trigger CICR, the propagation stops. This is indeed the case of cells A4 and A5. Cells were coupled by sigmoid gap junctions and experienced reflective boundary conditions. (1.60 MB TIF) [file pcbi.1000909.s003.tif]

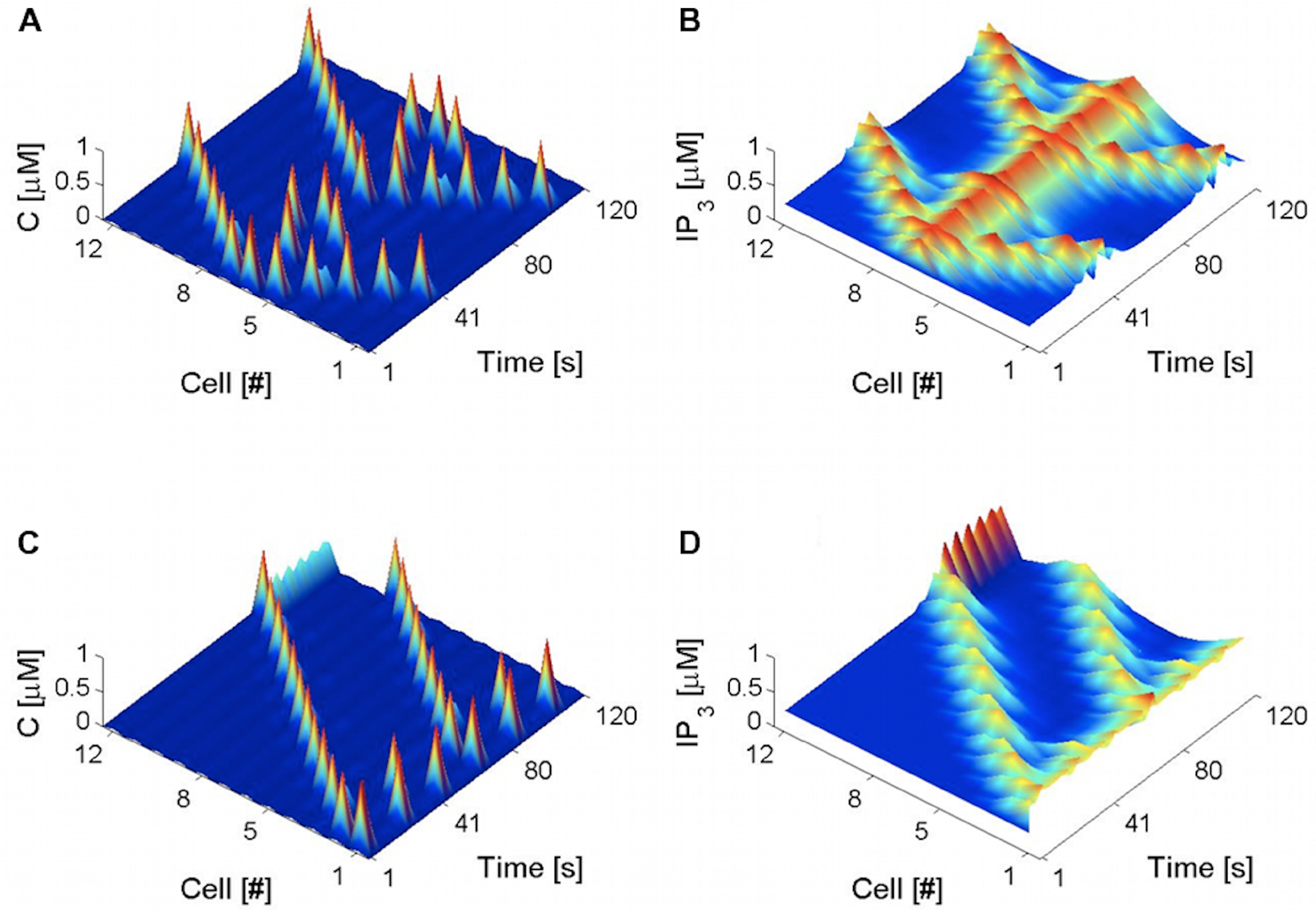

Supplement: Figure S4 — Propagation patterns with non-linear sigmoid-like gap junctions in an astrocyte chain of 12 FM-encoding cells with periodic (a,b) or absorbing (c,d) boundary conditions. Stimulation triggered by IP3 bias = 1.0 µM from t = 0 s to t = 120 s applied to the central cell (i.e. cell 6) (a,b) or the first cell in the chain (c,d). (5.58 MB TIF) [file pcbi.1000909.s004.tif]

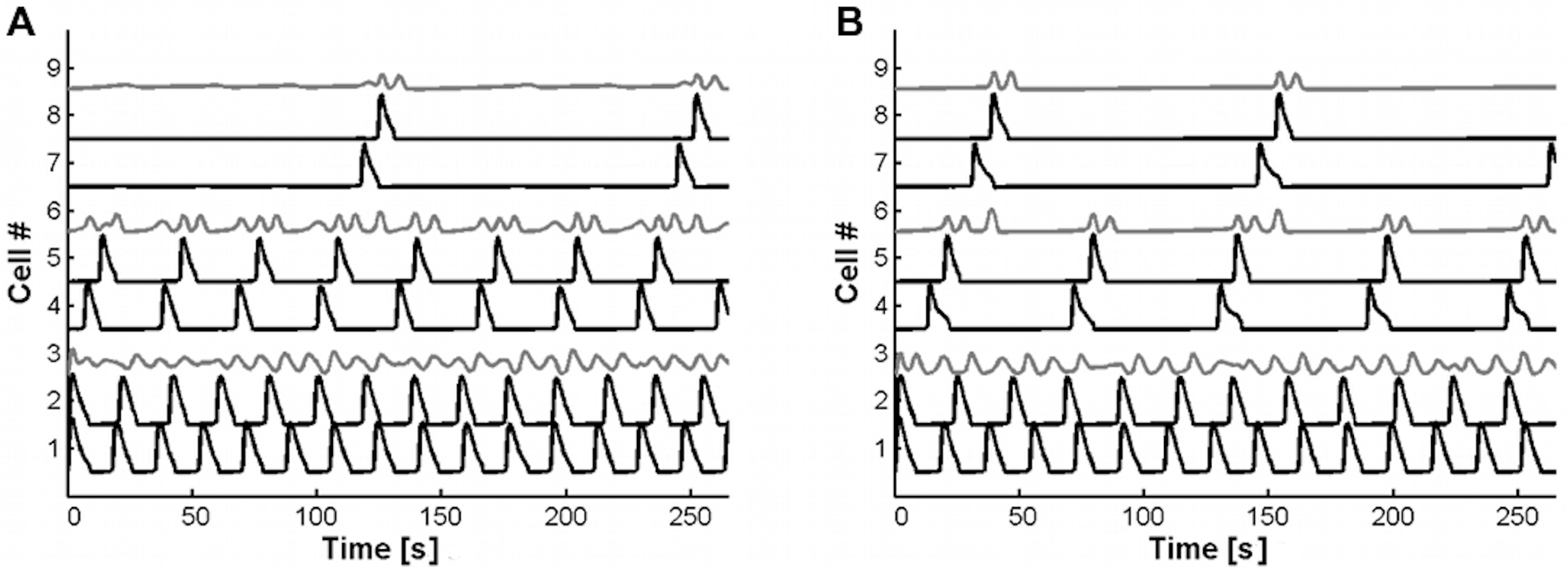

Supplement: Figure S5 — Calcium traces for wave propagation in composite astrocyte chains constituted of both FM (black) and AFM (gray) cells with (a) IP3 thr = 0.215 µM or (b) IP3 thr = 0.3 µM. The diffusion threshold is critical to determine the efficiency of transmission of Ca2+ waves along astrocyte chains. Other parameters as in Figure 7c. (2.04 MB TIF) [file pcbi.1000909.s005.tif]

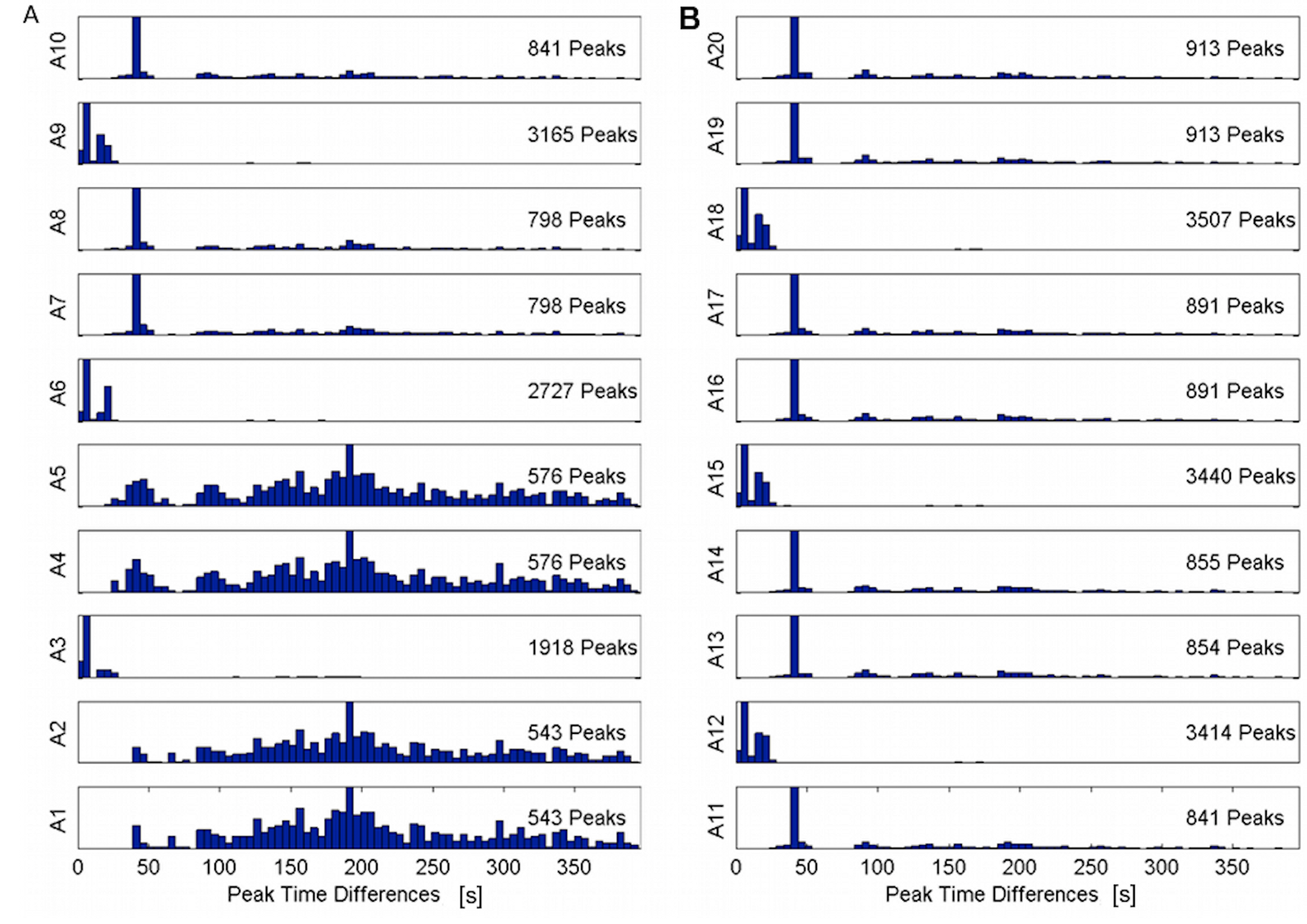

Supplement: Figure S6 — Interpulse interval distributions for the simulations shown in Figure 7 (main text). (a) Distributions for cells A1 to A10. (b) Distributions for cells A11 to A20. In several cells (e.g. cells A1, A2, A4 and A5), the distribution is broad, and large intervals are as commonly observed as smaller ones. Each panel indicates the number of pulses counted. (5.04 MB TIF) [file pcbi.1000909.s006.tif]
