# Supplementary material for: Nonlinear Gap Junctions Enable Long-Distance Propagation of Pulsating Calcium Waves in Astrocyte Networks
Source: PLoS Comput Biol. 2010 Aug 26;6(8):e1000909. doi: 10.1371/journal.pcbi.1000909 (PMC2928752; doi:10.1371/journal.pcbi.1000909)
Supplement: Table S1 — Parameter list and used values. The parameters of the Li-Rinzel core of the ChI model were taken according to previous studies [De Pittà et al. (2008) Phys Rev E 77: 030903(R); De Pittà et al. (2009) Cogn Proc 10:55 and De Pittà et al. (2009) J Biol Phys 35:38]. Gap junction parameters were chosen in order to allow sufficient diffusion of IP3 across cell borders in order to trigger CICR in neighboring cells. With these parameters, Ca2+ and IP3 oscillations in the simulations show amplitudes that are consistent with those reported in the literature [Politi et al. (2006) Biophys J 90:3120; Mishra and Bhalla (2002) Biophys J 83:1298]. When two values are indicated for the same parameter, the first one corresponds to AFM conditions, the second one to FM conditions. (0.05 MB DOC) [file pcbi.1000909.s007.doc]

| **Parameter** | **Value** (AFM/FM) | **Units** | **Description** |
| --- | --- | --- | --- |
| *Li-Rinzel core parameters* | | | |
| *C_0_* | 2.0 | μM | Total cell free [Ca^2+^] per cytosolic volume |
| *c_1_* | 0.185 | – | Ratio between ER and cytosol volumes |
| *r_C_* | 6.0 | s^-1^ | Maximal CICR rate |
| *r_L_* | 0.11 | s^-1^ | Maximal rate of Ca^2+^ leak from the ER |
| *v_ER_* | 0.9 | μM·s^-1^ | Maximal SERCA uptake rate |
| *d_1_* | 0.13 | μM | IP_3_ dissociation constant |
| *d_2_* | 1.049 | μM | Ca^2+^ inactivation dissociation constant |
| *d_3_* | 0.9434 | μM | IP_3_ dissociation constant |
| *d_5_* | 0.08234 | μM | Ca^2+^ activation dissociation constant |
| *a_2_* | 0.200 | s^-1^ | IP_3_R binding rate constant for Ca^2+^ inhibition |
| *K_ER_* | 0.10 / 0.05 | μM | SERCA Ca^2+^ affinity |
| *IP_3_ metabolism parameters* | | | |
| *v_δ_* | 0.12 / 0.7 | μM·s^-1^ | Maximal rate of IP_3_ synthesis by PLCδ |
| *K_PLCδ_* | 0.1 | μM | Ca^2+^ affinity of PLCδ |
| *κ_δ_* | 1.5 | μM | Inhibition constant of PLCδ activity |
| *v_3K_* | 4.5 | s^-1^ | Rate of IP_3_ degradation by 3K |
| *K_3K_* | 0.7 | μM | Half maximal degradation rate of IP_3_ by IP_3_-3K |
| *K_3_* | 1 | μM | Half-saturation constant for Ca^2+^-dependent IP_3_-3K activation |
| *r_5P_* | 0.04 / 0.21 | s^-1^ | Rate of IP_3_ degradation by IP-5P |
| *F* | 2 | μM·s^-1^ | Gap junction permeability (or coupling strength) |
| *Nonlinear gap-junction parameters* | | | |
| *IP_3_*^shift^ | 0.3 | μM | Half-maximal diffusion IP_3_ threshold |
| *IP_3_*^scale^ | 0.05 | μM | Slope factor |
| *IP_3_*^bias^ | 0.8 / 1.0 | μM | Imposed IP_3_ level in the stimulated cell |
