# Supplementary material for: Nonlinear Gap Junctions Enable Long-Distance Propagation of Pulsating Calcium Waves in Astrocyte Networks
Source: PLoS Comput Biol. 2010 Aug 26;6(8):e1000909. doi: 10.1371/journal.pcbi.1000909 (PMC2928752; doi:10.1371/journal.pcbi.1000909)
Supplement: Text S1 — Supplementary information text. (0.10 MB DOC) [file pcbi.1000909.s008.doc]

**Nonlinear gap junctions enable long-distance propagation of pulsating calcium waves in astrocyte networks**

Mati Goldberg, Maurizio De Pittà, Vladislav Volman, Hugues Berry and Eshel Ben-Jacob

**SUPPLEMENTARY INFORMATION**

1. **The *ChI* model for intracellular Ca^2+^ dynamics**

Intracellular calcium dynamics in cells are the result of an intricate signaling pathway that mostly rests on Ca^2+^ exchange between the cytoplasm and internal stores, such as the endoplasmic reticulum (ER) or mitochondria [1]. In astrocytes, Ca^2+^ exchange with the ER plays the prominent role [2] and the concentration of cytoplasmic Ca^2+^ depends on three distinct fluxes (illustrated in Figure 1a).

(1) Transfer of Ca^2+^ from the cytoplasm to the ER lumen is mediated by an active pump, the (Sarco-)Endoplasmic-reticulum Ca^2+^ ATPase (SERCA). SERCA contributes to the maintenance of higher Ca^2+^ concentrations in the ER than in the cytoplasm.

(2) This concentration gradient drives a passive Ca^2+^ leak from the ER to the cytoplasm.

(3) Specific Ca^2+^ transporters, called inositol triphosphate receptor-channels (IP_3_Rs, for short), contribute a third flux, transporting molecules from the ER to the cytoplasm. The regulation of IP_3_Rs is remarkable: their activity is upregulated by a small molecule, inositol triphosphate (IP_3_) and by small-to-intermediate cytoplasmic Ca^2+^ levels. However, large Ca^2+^ concentrations inhibit IP_3_R activity.

Therefore, a small increase in cytoplasmic Ca^2+^ levels activates IP_3_Rs, which leads to increased Ca^2+^ transport from the ER, ultimately increases cytoplasmic Ca^2+^. Because of this positive feedback, Ca^2+^ transport through IP_3_Rs is usually called Ca^2+^-induced Ca^2+^ release (CICR). At some point however, the concentration of Ca^2+^ within the cytoplasm becomes large enough to inhibit IP_3_R activity and the surge of Ca^2+^ through IP_3_R terminates. These three transport mechanisms are classically captured by a mathematical model due to Li and Rinzel [3-5], given by eq.(1) and eq.(2) in the article. As is obvious from these equations, the Li-Rinzel (LR) model has two dynamical variables: *C*, the free cytosolic Ca^2+^ concentration and *h,* the fraction of inactive (closed) IP_3_R channels. The concentration of IP_3_ in the cytoplasm (*IP_3_*) instead is a parameter. To simulate this system, one must specify the mathematical formulation of the three fluxes *J*_pump_, *J*_leak_ and *J*_chan_ as well as those of the steady fraction of closed IP_3_Rs, *h*_∞_ and of the channel open/close transition time constant, *τ_h_*. In the present work, we have used the classical formulations originally introduced by Li and Rinzel [3-5].

As mentioned above, one feature of the Li-Rinzel model is that it treats the concentration of IP_3_ as a mere parameter, i.e. *IP_3_* in equations (1-2) is a constant value in time and does not change when the dynamical variables *C* and *h* vary over time. Yet, inspection of the intracellular Ca^2+^ signaling pathway (Figure 1b) immediately shows that IP_3_ concentration in the cytosol is coupled to Ca^2+^ dynamics, and therefore is expected to change with *C*. In particular, IP_3_ formation and degradation are both regulated by cytoplasmic Ca^2+^.

We recently presented a model called the *ChI* model, that basically extends the Li-Rinzel model but includes IP_3_ as a third dynamical variable [5]. To this aim, we had to simplify the complexity of the IP_3_ signaling pathway (Figure 1c, d). This allowed us to derive a tractable yet realistic model. For clarity, we briefly describe the *ChI* model below. Further details and analyses can be found in [5].

In astrocytes, endogenous IP_3_ dynamics (Figures 1c,d) mainly result from three enzymatic processes [6-8]. Endogeneous IP_3_ is produced from a membrane lipid called phospatidylinositol biphosphate (PIP_2_) in a reaction catalyzed by phospholipase Cδ (PLCδ). It can be degraded through phosphorylation (catalyzed by IP_3_ 3-kinase, IP_3_-3K) or through dephosphorylation (catalyzed by inositol polyphosphate 5-phosphatase, IP-5P). Experimentally, PLCδ is activated by cytoplasmic Ca^2+^ in a cooperative fashion with a Hill exponent close to 2. But PLCδ is also inhibited by IP_3_ concentrations larger than circa 1 µM. We thus expressed IP_3_ production through PLCδ as:

$P_{PLC\delta}(C,IP_{3})=v_{\delta}\frac{K_{I}}{K_{I}+IP_{3}}\text{Hill}(C^{2},K_{PLC\delta})$ (S1)

where *ν_δ_* is the maximal rate of IP_3_ production by PLCδ, and we define generic Hill functions as $\text{Hill}(x^{n},K)\equiv x^{n}/(x^{n}+K^{n})$. Dephosphorylation of IP_3_ by IP-5P displays pure Michaelis-Menten kinetics, but its Michaelis constant (≈10 µM) is higher than physiological IP_3_ levels. It can thus be expressed as a linear reaction term:

$D_{5P}(IP_{3})=r_{5P}IP_{3}$ (S2)

As evidenced in Figure 1d, the regulation of IP_3_ 3-kinase by cytoplasmic Ca^2+^ is a complicated mechanism and includes interactions between Ca^2+^, calmodulin and CaMKII. Using the algebraic properties of Hill functions however, we have been able to lump all this complexity into the product of a 4^th^-order Hill function of Ca^2+^ with a first-order Hill function of IP_3_ (see [5] for further explanation):

$D_{3K}(C,IP_{3})=v_{3K}\text{Hill}(C^{4},K_{D})\text{Hill}(IP_{3},K_{3})$ (S3)

Taken together, the IP_3_ concentration in the *ChI* model is a third dynamical variable, whose dynamics are given by:

$\frac{dIP_{3}}{dt}=P_{PLC\delta}(C,IP_{3})-D_{3K}(C,IP_{3})-D_{5P}(C,IP_{3})+v_{bias}$ (S4)

where *v_bias_* defines an external input of IP_3_ coming e.g. from a neighboring astrocyte cell coupled through gap junction. Note that equation (S4) yields to equation (3) in the article when *v_bias_*=0. Equations (1-2) and (S1-S4) together define the *ChI* model of intracellular Ca^2+^ dynamics.

1. **Bifurcation analysis of the *ChI* model and encoding modes**

*a) Isolated cell*

We start our analysis with a description of the dynamics of the uncoupled case, i.e. the *ChI*model equations (1-2) and (S1-S4). Figure S1 shows bifurcation diagrams when the input bias of IP_3_ varies in equation (S4). For either small or large biases, the IP_3_ production term in equation (S4) is balanced by the two degradation terms. This gives rise to two stable steady-(time independent) states (black thick lines in Figures S1a, b). Further analysis of equations (1-2, S1- S4) shows that between the two steady-state regimes, spontaneous oscillations of the three variables (*C*, *h* and *IP_3_*) arise. The evolution of the envelop of these oscillations (minimal and maximal values during the cycle) with *ν_bias_* is shown in 3D plots as thin gray lines (Figures S1a, b) or in 2D projections as full thick lines (Figures S1c, d).

The general scenario indicated above (i.e. low steady state then oscillations then high steady state as *v_bias_* increases) is qualitatively conserved when the cell parameters vary in realistic ranges. However, a key observation for the present work is that, depending on the value of some cell physiological parameters (e.g. the affinity of SERCA pumps), the *nature* of the obtained oscillatory behavior changes. The major change concerns the type of bifurcation that is associated with the birth of the oscillatory behavior. Oscillations can arise through a Hopf bifurcation (Figure S1a) or through a Saddle-Node-on-Invariant-Circle (SNIC) bifurcation (Figures S1b). Now, the nature of the bifurcation decides how a change in the incoming IP_3_ bias is encoded in the Ca^2+^ oscillations. In fact one can classify these encoding behaviors by three classes.

(1) The amplitude of Ca^2+^ oscillations more than doubles across the whole oscillatory range while their frequency stays almost constant. We refer to this behavior as the amplitude-modulation (AM) encoding mode.

(2) The frequency of Ca^2+^ oscillations more than doubles across the whole oscillatory range while their amplitude stays almost constant. We refer to this behavior as the frequency-modulation (FM) encoding mode.

(3) Both the amplitude and the frequency of Ca^2+^ oscillations change by more than a factor of 2 across the whole oscillatory range. We refer to this mode as the mixed amplitude-frequency-modulation (AFM) encoding mode.

Note that the waveform of the oscillations is also affected by the difference of the bifurcation type: FM-type oscillations usually display pulse-like waveforms, whereas AM-type ones are closer to sinusoidal [5].

In Figure S1 we illustrate the encoding modes displayed by the *ChI* model. The left-hand column (Figures S1a, c, e) illustrates a typical AFM behavior: increasing the IP_3_ input bias yields an increase of the oscillation amplitude of *IP_3_* and *C* oscillations (Figure S1c) but also a marked decrease of their period (Figure S1e). The situation is more complex for the parameters used in the right-hand column: here the period varies dramatically but the amplitude of intracellular Ca^2+^ oscillations is close to constant (though the amplitude of *IP_3_* oscillations varies). This corresponds to a FM regime (at least for *C*). Furthermore, as Ca^2+^ transport through the SERCA pumps is larger for FM conditions [9], the range of IP_3_ bias for which oscillations are predicted, is much narrower in the AFM regime than in the FM one.

*b) Coupled cell network*

We now consider a linear chain made of *N* *ChI* cells coupled to their two nearest neighbors by gap junctions as defined by equations (7-13). In the case of nonlinear coupling at least, one could expect that the dynamics of the cells would be different from the behavior predicted in the uncoupled (isolated) case studied above. To evaluate these changes, we first computed bifurcation diagrams for the coupled system (while Figure S1 shows bifurcation diagrams for isolated cells).

Unfortunately, deriving such bifurcation diagrams is arduous and uncertain from a numerical point of view because the resulting system is strongly nonlinear. As a consequence, the corresponding diagrams contain imperfectly resolved parts (see Figure S2 caption). Figure S2 shows bifurcation diagrams for *N*= 7 FM-encoding cells coupled by sigmoid-like gap junctions with reflective boundaries. We apply the IP_3_ stimulus on the central cell in the chain (namely Cell 4 in Figure S2). In this case, we can consider only half of the chain and plot bifurcation diagrams for cells 4 to 7 only, since for symmetry reasons cells 3 to 1 display the same behavior as cells 5 to 7.

The bifurcation diagram of the central cell is qualitatively similar to the uncoupled case (compare Cell 4 in Figure S2 with Figures S1b, d). In particular, oscillations still arise through a SNIC bifurcation (at *IP_3_*^bias^ ≈ 0.72 µM). On the other hand, the bifurcation diagram beyond the SNIC displays multiple period-doubling cascades as well as different co-existing stable oscillatory regimes. As a consequence, the oscillatory regime in the central cell rapidly becomes extremely complex. The bifurcation diagrams for the other cells are also globally similar to that of the central (stimulated) cell, except that the fixed-point branches tend to get more horizontal when the distance from the stimulated cell increases. Furthermore, qualitatively similar diagrams are also obtained for different boundary conditions as well as for different stimulus locations (results not shown).

In general, these diagrams show FM pulse-like oscillatory regimes at low *IP_3_*^bias^ values, which can turn into complex oscillations for larger *IP_3_*^bias^ values. Yet because stable oscillation regimes can coexist in the bifurcation diagrams with stable fixed points, it cannot be predicted from these diagrams whether an IP_3_ input arriving to a given cell from its neighbor will trigger pulse-like oscillations or not, i.e. whether it will switch the system from the fixed point to the oscillatory regime. Accordingly, we used extensive numerical simulations to investigate under what conditions one could observe intercellular propagation of Ca^2+^ waves along the astrocyte chain.

In agreement with previous studies (see [10] for a review), IP_3_-triggered CICR indeed allows intercellular Ca^2+^ wave propagation in our modeling framework, as shown in Figure S3. Here the simple case of *N = 5* FM *ChI* astrocytes coupled through sigmoid gap junctions is considered and the first cell (*cell A1*) is stimulated from *t* = 10 s to *t* = 30 s by a constant bias of *IP_3_*^bias^ = 0.8 µM. In agreement with the bifurcation diagrams shown in Figure S2, pulse-like Ca^2+^ oscillations are observed. Note that the 20-second long stimulation used here is shorter than the predicted period of oscillations at such *IP_3_*^bias^ value. Therefore, only a single pulse is observed. In general when a large-enough IP_3_ pulse arrives to a given cell, either by direct external stimulation or from a neighbor coupled cell, CICR first generates a neat pulse-like Ca^2+^ response (Figure S3, *black curves*), which predates a pulse-like increase of IP_3_ (Figure S3, *dashed blue curves*). Part of this IP_3_ then diffuses to the neighbor cells in the form of a new pulse. Therefore, as long as the newly formed IP_3_ pulses are large enough, IP_3_-triggered CICR is preserved and a propagating pulsating Ca^2+^ wave, mediated by IP_3_ diffusion, is observed.

On the contrary, lack of IP_3_ stops wave propagation, as it happens for example in Figure S3 for the IP_3_ pulse that leaves cell A3 for cell A4. This pulse is indeed too small to trigger CICR in cell A4, so that Ca^2+^ wave propagation ceases beyond cell A3. Notably, the distance (in terms of number of cells) that a Ca^2+^ wave can travel along the chain strongly depends both on the nature of the gap-junction coupling and on cell type (AFM or FM) as is shown in the article.

**References**

1. Berridge MJ, Lipp P, Bootman MD (2000) The versatility and universality of calcium signalling. Nat Rev Mol Cell Biol 1: 11­21.
2. Agulhon C, Petravicz J, McMullen AB, Sweger EJ, Minton SK, et al. (2008) What is the role of astrocyte calcium in neurophysiology? Neuron 59: 932­946.
3. Li Y, Rinzel J (1994) Equations for InsP_3_ receptor­mediated [Ca^2+^]_i_ oscillations derived from a detailed kinetic model: A Hodgkin­Huxley like formalism. J Theor Biol 166: 461­473.
4. De Pittà M, Goldberg M, Volman V, Berry H, Ben­Jacob E (2009) Glutamate­dependent intracellular calcium and IP_3_ oscillating and pulsating dynamics in astrocytes. J Biol Phys 35: 383­411.
5. De Pittà M, Volman V, Levine H, Pioggia G, De Rossi D, et al. (2008) Coexistence of amplitude and frequency modulations in intracellular calcium dynamics. Phys Rev E 77: 030903(R).
6. Rebecchi MJ, Pentyala SN (2000) Structure, function, and control of phosphoinositide­specific phospholipase C. Physiol Rev 80: 1291­1335.
7. Sims CE, Allbritton NL (1998) Metabolism of inositol 1,4,5­trisphosphate and inositol 1,3,4,5­tetrakisphosphate by the oocytes of Xenopus laevis. J Biol Chem 273: 4052­4058.
8. Irvine RF, Letcher AJ, Heslop JP, Berridge MJ (1986) The inositol tris/tetrakisphopshate pathway – demonstration of Ins(1,4,5)P_3_ 3­kinase activity in animal tissues. Nature 320: 631­634.
9. De Pittà M, Volman V, Levine H, Ben­Jacob E (2009b) Multimodal encoding in a simplified model of intracellular calcium signaling. Cogn Proc 10: 55­70.
10. Falcke M (2004) Reading the patterns in living cells ­ the physics of Ca^2+^ signaling. Adv Phys 53: 255­440.
